# Effects of oral contraceptives and natural menstrual cycling on environmental learning

**DOI:** 10.1186/s12905-018-0671-4

**Published:** 2018-11-07

**Authors:** Filippo Bianchini, Paola Verde, Stefano Colangeli, Maddalena Boccia, Felice Strollo, Cecilia Guariglia, Giuseppe Bizzarro, Laura Piccardi

**Affiliations:** 1 Department of Psychology, Sapienza University of Rome, Rome, Italy; 2 Aerospace Medicine Department, Italian Air Force, Experimental Flight Centre, Pratica di Mare, Pomezia (RM), Italy; 3 Department of Physiology and Pharmacology, Sapienza University of Rome, Rome, Italy; 4 Neuropsychology Unit, IRCCS Santa Lucia Foundation, Rome, Italy; 5 Department of Pharmacology and Biomolecular Sciences, Milan University, Milan, Italy; 6 Department of Life, Health and Environmental Sciences, L’Aquila University, L’Aquila, Italy

**Keywords:** Navigational memory, Exogenous hormones, Spatial orientation, Sex differences, Menstrual cycle

## Abstract

**Background:**

Endogenous ovarian hormones as well as exogenous oestradiol and progesterone play an important role in cognitive processing. Specifically, these hormones play a role in different aspects of memory, both in terms of storage capacity and temporal duration of the mnemonic track. These hormones also have various effects on different types of memory (i.e., verbal, visuo-spatial, prospective). This study investigated the effects of hormones on topographic memory, a type of memory specifically needed to recall a pathway and to acquire spatial information about locations, distances, and directions.

**Methods:**

We compared 25 naturally cycling women (NCW) in two different cycling phases, the early follicular phase (4th - 5th days) and the mid-luteal phase (20th-21st days), with 26 women taking oral contraceptives (OC) tested in the active pill phase (20th to 21st day of OC cycle) and the inactive pill phase (2nd to 4th day of OC cycle). Both groups performed the Walking Corsi Test to assess topographic memory in their respective cycling phases. Women were instructed to learn an eight-step sequence path and recall the path five minutes later.

**Results:**

We found that the two groups differed in terms of learning the 8-step sequence path; OC users were always better (4–5 days vs. 20–21 days) than NCW. No differences emerged in the delayed recall of the same path.

**Conclusions:**

As already observed in other memory domains (i.e., verbal memory, emotional memory), OC users showed an advantage in terms of topographic learning. Our results might be explained by hormonal mechanisms and may suggest the future application of OC in women with topographic disorders or visuo-spatial difficulties.

## Background

Ovarian hormones are pivotal for the physiological maintenance of brain function. There is growing evidence attesting to the relevance of endogenous ovarian hormones as well as exogenous oestradiol and progesterone in cognitive processing. It is known that the menstrual cycle phase and hormonal changes due to pregnancy or other medical conditions may modulate memory quality (e.g., [1, 2]). However, the debate is still open as to the types and aspects of memory affected, such as temporal duration (working memory and long-term memory) and storage capacity. In this view, menstrual cycle studies offer a non-invasive approach for investigating cognitive changes related to oestrogen endogenous fluctuations. Women were generally asked to perform cognitive tests at different menstrual cycle phases, coinciding with minimal and maximal gonadal hormone secretions. For example, one test was performed when oestradiol and progesterone levels were at nadir levels (early follicular phase, days 2nd**–**5th), and the other test was performed when oestradiol and progesterone levels were high (midluteal phase, days 19th**–**24th). Since the early 1990s, verbal articulation and speeded manual skills have been shown to improve during cycle phases characterized by high levels of oestrogen and/or progesterone (midluteal phase), whereas visuospatial skills are improved during the follicular phase of the cycle [3, 4]. During the mid-luteal phase, an enhancement of verbal implicit memory was also reported [5], as was delayed recall of geometric figures [6].

In oral contraceptive (OC) users, there is a 7-day, hormone-free interval of inactive placebo pills, during which the pituitary ovarian axis slowly regains activity [7]. This process begins with an increase in follicle-stimulating hormone (FSH) levels, which leads to oestradiol production. Oestradiol levels have been found to remain suppressed until day 6 of the inactive placebo phase (i.e., day 27 of the cycle), when they become significantly greater than levels during the active pill phase [8]. Few studies have compared the different phases in OC users, despite the possibility of the active and inactive phases of OC producing different effects on cognitive tasks, especially memory tasks. Grinspoon et al. [9] found no effects on performance after 3 months of treatment with a triphasic OC, as measured by the Wechsler Memory Scale-Revised or the Complex Figure Recall Test in women tested during unspecified cycle days. Interestingly, in OC users, the negative effects of cortisol on memory seem to be reduced [10]. This finding is mainly due to an interaction between hypothalamic-pituitary-adrenal stress-related hormones and hypothalamic-pituitary-gonadal hormones (see [11]). In line with this result, Petersen et al. [11] found a reduction in false memory susceptibility, while Nielsen et al. [12] observed better recall of emotional gist in OC users. Gogos et al. [13] found that OC users performed better on verbal memory tasks compared to NCW. This finding was replicated by Mordecai et al. [14], who found that NCW did not show changes in verbal memory across the cycle, whereas OC users showed enhanced memory during the active pill phase. However, these authors did not find any effect on visuospatial memory, verbal fluency, visuospatial abilities or attention. Generally, the positive effect of OC has not been systematically found for visuo-spatial cognition, and, when evidenced, it seems to be related to the type of OC used [13–16]. To the best of our knowledge, no studies have attempted to assess the effect of OC on topographic memory, despite findings demonstrating it to be sensitive to gender-related effects and hormonal fluctuations [2, 17–29]. Due to the expected effect of OC on memory, and due to the presence of gender-related effects on topographic memory, this type of memory could be a target of OC effects. Topographic memory is a special type of memory that allows people to recall a pathway from memory. When we move through the environment, we acquire spatial information about location, distance and direction, which we store in memory for future retrieval when we try to reach a familiar place or give directions about its location to others. Topographic memory is also needed to recall and figure out a route or a shortcut to reach a place, as well as to recognize a previously visited location. This mental process requires not only visuospatial information but also vestibular and proprioceptive information relative to whole body movements, together with memory for locations (e.g., [20, 23, 30, 31]).

This memory allows us to learn new environments [20, 30–33]. A body of mounting evidence attests to the presence of gender differences in topographic memory, showing that men perform better than women (e.g., [22–34]). Specifically, women were found to be slower in learning new environments compared to men, whereas they showed no difficulties in retrieving environmental information from long-term storage [2, 20–22]. Several hypotheses have been formulated to explain gender differences in spatial memory, including biological factors such as hormones (e.g., [7]; for a review [34]). It is known that progesterone and oestradiol modulate neural activity in the hippocampus, the amygdala and the prefrontal cortex (see [35] for review). Endogenous spontaneous fluctuations of oestradiol and progesterone in different menstrual cycle phases have been found to differently affect brain activity during 3-D mental rotation. Specifically, 3-D mental rotation produced larger activation of the temporo-parietal network during the mid-luteal phase, whereas it produced greater activation of the superior temporal gyrus and medial frontal gyrus during the early follicular phase [36]. Interestingly, the temporo-parietal network is involved in spatial navigation and memory [37–40]. Therefore, endogenous fluctuations in hormonal levels across the menstrual cycle may affect performance on memory tasks. OCs are the most commonly prescribed medications in women of reproductive age. OCs dampen endogenous fluctuations in ovarian hormones. Indeed, all OCs act through negative feedback on the hypothalamic pituitary gonadal axis, thus leading to low endogenous sex hormones. Triminulet® was chosen as the OC preparation for our study because of its triphasic nature, allowing it to deliver synthetic sex hormones called EE2 (ethynyl estradiol) and gestodene for 21 days at three different doses. Such dosages were designed to somehow mimic (while inhibiting) cyclic ovarian function by levelling down endogenous oestradiol and providing the organism, including the hypothalamus, with a low, semi-constant ethynyl estradiol level and a stepwise increase in the progesterone-like signal [41]. It is also worth pointing out that gestodene, the progestin contained in the pill chosen for our study, lacks both any androgenic and anti-androgenic effects. However, the more widely used biphasic pills would have acted through an ovarian suppressive mechanism that is less associated with cyclic brain exposure to oestradiol and progesterone oscillation patterns. Behavioural effects may be observed due to the use of OC. Interestingly, brain activity within a fronto-parietal network has been found to differ across OC users and freely cycling women in early follicular and mid-luteal phases (for review, see [35]).

This study investigated the effect of endogenous and exogenous hormonal fluctuations on topographic memory. To this aim, we compared women taking OC with naturally cycling women (NCW). We also assessed the hormonal effects in different stages of the menstrual cycle and, to this purpose, we tested women in two different cycling phases: the early follicular phase (4th–5th day of the natural menstrual cycle), characterized by mid-low levels of oestradiol and almost null progesterone secretion, and the mid-luteal phase (20th-21st day of the menstrual cycle), characterized by higher yet unpredictably variable levels of oestradiol and high levels of progesterone.

Since hormonal fluctuations are controlled in OC users [42], and, given the well-known effects of sex steroids on memory performance, we hypothesized that OC users would perform better than NCW. To this end, we adopted the Walking Corsi Test (WalCT: [20, 23]), an experimental tool aimed at assessing topographic memory, and repeatedly found it to be sensitive to gender differences [2, 20, 22, 23, 27, 43].

## Method

### Participants

Fifty-one healthy women (26 OC users; 25 NCW) who were students from the “Sapienza” University of Rome took part in this study (OC users’ mean age: 24.7; SD: 2.6; NCW’s mean age: 25.7; SD: 3.1; no significant differences between the two groups: **t**-value: 1.348; ***p*** = 0.184). The participants were first screened by phone for general inclusion criteria. OC users were recruited by general practitioners who gave only the names of those women taking the pill as a contraceptive. The following “exclusion criteria” proposed by Crook et al. [44] were applied during enrolment: history of head trauma, any neurological or psychiatric illness, history of brain fever or any other state of altered consciousness, use of benzodiazepines in the previous 3 months, use of illicit drugs, any visual, auditory or motor impairment, any symptomatic cardiovascular conditions, breathing problems, or diseases capable of causing cognitive impairment. Considering the aim of the study, we also asked participants for the presence of endocrine pathologies. NCW reported a regular menstrual cycle (average length 28 ± 4 days) [45]. All participants completed the Familiarity and Spatial Cognitive Style Scale (FSCS: [46]) to exclude the presence of navigational difficulties or developmental topographical disorientation [47, 48]. All women had normal or corrected-to-normal vision. The local ethics committee (Psychology Department of “Sapienza” University of Rome) approved this study. All volunteers gave their written informed consent to participate in the experiment and to be re-contacted for the second testing phase. OC users were already taking contraception before the time of the study (start assumption time ranged from 6 months to 3 years). We included only participants that used a triphasic pill (i.e., Triminulet®: gestodene 0.050 mg + EE2 0.03 mg for 5 days, gestodene 0.070 mg + EE2 0.04 mg for 4 days, gestodene 0.100 mg + EE2 0.03 mg for 10 days) because, as stated above, it was more suitable to our study due to its more physiologic hormonal release pattern and effective pharmacologic ovarian suppression [49].

### Materials and procedure

**Fig. 1:**
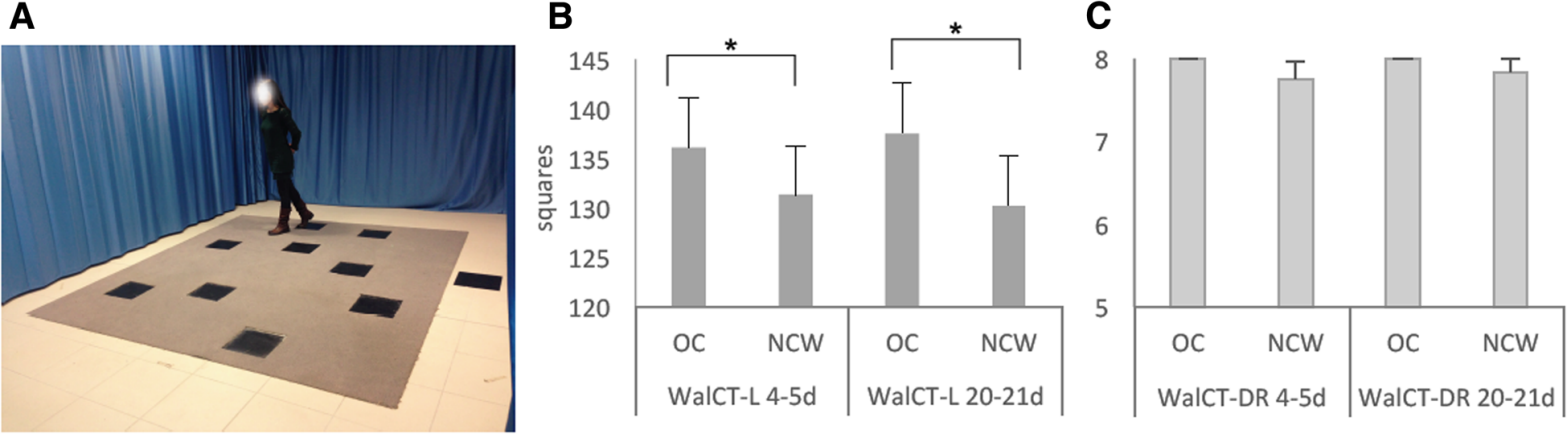
**a** Experimental set-up. Written informed consent was obtained from the subject (M.B., one of the authors) represented in the picture. **b** Means and standard errors of scores on learning the WalCT (y-axis) by OC users and NCW in the early follicular phase (4th - 5th day) and the mid-luteal phase (20th-21st day) (x-axis). **c** Means and standard errors of scores obtained on delayed recall of WalCT (y-axis) by OC users and NCW in the early follicular phase (4th - 5th day) and the mid-luteal phase (20th-21st day) (x-axis)

The WalCT test [20, 23] is a larger version of the Corsi Block-Tapping Test (CBT [50]: 3 × 2.5 m; scale 1:10 of the CBT). Nine black squares (30 × 30 cm) were placed on the floor of an empty room in the same position as in the standard CBT. In addition, one more square (starting point) was placed 50 cm from the line to mark the perimeter of the walking area (Fig. 1a).

During the learning phase, the examiner showed an eight-square path by walking on squares at a rate of one square per 2 s. The participants were instructed to learn the path. The learning criterion was reached if participants reproduced the correct path three times in a row. If participants failed to reproduce the path, the examiner would show it again. The number of correct black squares reproduced in each trial was computed for the final score, but no feedback about correctness was provided. The learning criterion (indicating that learning was achieved) corresponded to three consecutive correct sequence reproductions. If the participant did not achieve the learning criterion, the sequence was repeated for a maximum of 18 trials. The learning score was calculated by giving one point for each square correctly guessed until the criterion was achieved. The score corresponding to the correct performance of the remaining trials was added to this score (max number of trials = 18; maximum total score: 144).

Once the sequence was learned, the participants spent five minutes completing the FSCS and the anamnesis questionnaire, including general questions about demographic and health details. The participants were then asked to reproduce the previously learned eight-square walking path. The delayed recall score was calculated as the number of squares correctly reproduced (maximum score = 8).

For NCW, testing was scheduled during the early follicular phase (2nd–5th day after menses onset) and mid-luteal phase (20th-21st day after menses onset). The OC users were tested during both the low progestogen pill phase (4th–5th day, where day 1st is defined as the first day of the low-dose pill series) and the high progestogen pill phase (20th-21st day of the OC cycle), when the progesterone signal had progressively increased and reached a plateau at levels similar to those expected in NCW. Topographic memory in both groups was tested through parallel versions of the WalCT test (as in [2]). To avoid any facilitation effect due to knowledge of the experimental set-up, the order of the phase (follicular and luteal phases for NCW and the active and inactive pill phases for OC users) in which participants were tested was counterbalanced across participants. Specifically, 10 OC users and 10 NCW were tested during the mid-luteal phase (active pill phase) and then during the early follicular phase (inactive pill phase); the remaining participants were tested in the opposite order.

## Results

We performed 2 repeated-measures two-way ANOVAs (2 × 2) on the accuracy of learning performance and delayed recall on the WalCT with the two phases (4th–5th day vs. 20th-21st day) as repeated measures. A first repeated-measures two-way ANOVA was run with group (OC users vs. NCW) as the independent variable and performance on learning the WalCT during the two phases (4th–5th day vs. 20th-21st day) as repeated measures. A second repeated-measures two-way ANOVA was conducted with group (OC users vs. NCW) as the independent variable and performances on delayed recall WalCT during the testing time (4th–5th day vs. 20-21st day) as repeated measures.

The ANOVA carried out on learning the WalCT revealed a main ‘group’ effect [F(1,49) = 5.14, *p* = 0.02; partial η2 = 0.095] and the absence of a main effect for testing time [F(1,49) = 0.01, *p* = 0.91; partial η2 = 0.00]. The Group x Testing Time interaction was not significant [F(1,49) = 0.47, *p* = 0.50; partial η2 = 0.009]. This analysis showed that women who were taking OC learned the path in fewer trials than did NCW, which was true in both testing phases. The means and SDs of the two groups in the two testing phases are shown in Fig. 1b.

In contrast, the repeated-measures two-way ANOVA conducted on delayed recall of the WalCT did not show any significant main effect [Group: F(1,49) = 1.281, *p* = 0.26; partial η2 = 0.042 and Testing Time: F(1,49) = 2.172, *p* = 0.15; partial η2 = 0.04]. The same pattern was observed for the Group x Testing Time interaction: [F(1,49) = 2.172, *p* = 0.15; partial η2 = 0.04]. This finding means that once NCW had learned the sequence, they did not forget it, and their performance was comparable to that of the OC users in both testing phases.

## Discussion

In the present study, we investigated the effect of oral contraceptives and menstrual cycle phase on topographic memory. To our knowledge, no studies have investigated the effects of endogenous (i.e., fluctuations within the menstrual cycle) and exogenous oestradiol (i.e., the synthetic product administered via OC use) on topographic memory at two time points (days 4th**–**5th and days 20th**–**21st) in consecutive menstrual cycles. In our study, we found that women taking oral contraceptives performed better in terms of learning the WalCT than did NCW in both examined phases. Interestingly, the two groups were significantly different, and OC users learned the 8-step sequence in fewer attempts than did NCW. This finding is in line with the finding that those who ever used hormonal contraceptives perform significantly better than those who have never used in the domains of visuo-spatial ability as well as speed and flexibility with duration-dependent increases in performance [51].

We currently know that oestradiol treatment enhances hippocampal-dependent memory in both rodents and humans, and, at the low doses contained in any of the presently available pills, it promotes dopamine release in dopamine-terminal areas, including the caudate, prefrontal cortex, and nucleus accumbens; consequently, it promotes place memory. It has also been shown that progestin receptors are oestrogen-inducible and expressed in non-nuclear sites of hippocampal neurons ([52, 53]; see also review [54]). Indeed, memory formation and recall are complex processes that encompass two distinct long-term memory phases involving the hippocampus, amygdala and adjacent cortical medial temporal lobe (MTL) structures via a whole series of genomic (nuclear receptor dependent) and non-genomic (cell-membrane receptor dependent) intricate nested effects. These effects have been extensively described in an accurate review [55]. In our results, we found positive effects of pill use only on memory formation and not on later recall. Some explanations for this finding could include, first, that it is possible that the 8-step path we asked participants to learn was too easy, and, once learned, participants’ performance was almost at ceiling. However, examining our data does not support this interpretation since there is enough variability to exclude a ceiling effect. Second, as demonstrated by Saloner et al. [56], the delayed recall length for episodic memory may be important for highlighting differences in performance. Indeed, the delayed recall length usually used in experimental settings ranges from 5 to 30 min, but Saloner et al. [56] demonstrated that experiments that use longer delays (e.g., 1-week) are more sensitive to detecting differences. This finding is in agreement with the knowledge that the neurobiological process of memory consolidation extends well beyond the experimental time intervals, raising the possibility that tests using shorter delays (such as the WalCT) might not be fully sensitive to detecting subtle neurocognitive changes produced by OC use. In addition, the learning phase seems to be more sensitive to gender differences than the following retrieval phase, as also evidenced in other studies with other memory paradigms and longer delays (e.g., [21, 22, 57, 58]). Putting this evidence together, we can speculate that learning is more sensitive to individual differences. In light of this evidence, the pill may foster the acquisition of novel information, thus resulting in better learning performance. Once learning is achieved, no more effects of pill may be detected, as is seen with individual differences.

In the present study, we compared only OC users with NCW. Therefore, we can only state that OC users are better than NCW in memory formation in both phases of the cycle, suggesting a normalization of the effect of pill use. However, other studies that compared men and women in terms of sex-sensitive tasks during the different phases of women’s menstrual cycles found that OC users perform in a similar manner to men during their menstrual phase. Specifically, Moody [59] compared OC users and NCW on a mental rotation task (MRT) and failed to find any significant difference between them. Instead, they found a significant effect of menstrual phase and luteal phase. Furthermore, these authors showed that women performed similarly to men during their menstrual phase on this task. No further differences between OC and men have been reported. This issue was directly investigated by Wharton et al. [15], who tested whether the androgenic activity of OC mediates performance on sexually dimorphic cognitive tasks (such as mental rotations). These authors found an effect of the level of the androgenic component of the pill, with better performance in women who took more androgenic pills, such as second-generation pills. We did not compare women taking different generations of pills, but we had participants using a third-generation pill and found a similar pattern for a topographic learning task. We do not know if we would find other effects by comparing different generations of pills, but we can speculate that this effect is due to the androgenic component of the pill. In the future, it would be of interest to investigate potential mediating cognitive effects of different OC brands to assess the role of androgenicity in topographic memory.

### Limitations

Despite the fact that our results are novel and interesting, the present study has several weaknesses. First, the sample size was limited due to our choice of a “between-subjects” design to study menstrual and pill phase effects. Second, the results cannot be generalized to other pills, in particular those containing different compounds, e.g., mono- or biphasic pills. In addition, the lack of a baseline assessment before OC use in OC users may prevent any definitive conclusion about the causal role of OC in topographic memory. To overcome this limit, we are planning a future study where participants will be tested before taking the pill and then, several months after starting to use OC. However, in this case, the long follow-up time may limit availability to take part in the study. Third, we did not collect salivary samples to measure hormonal fluctuation, but we assumed these fluctuations on the basis of menstrual and pill phases. The absence of these physiological measures reduces the control of hormonal fluctuations. Indeed, our finding awaits replication with a larger number of participants, which would also include hormone level measurements. Fourth, the absence of other tests assessing different memory domains prevented us from considering this result as an advantage specific to topographic memory. We cannot exclude that OC users may also be better at verbal or visuo-spatial memory tasks compared to NCW, as partially supported by previous studies. However, taken in combination with previous results, our data shed some light on the memory domains influenced by OC.

Fifth, although we did not find group differences in delayed recall, this finding could be because the performance was maximized in both groups. A more complex task that allows greater variability in the results should be administered to rule out the absence of differences due to a possible ceiling effect. However, to our knowledge, gender differences generally come up during the learning phase rather than in the recall of already consolidated material. Therefore, we are confident in the present findings.

Finally, we did not compare performance by women and men on the WalCT, despite evidence of gender differences, with men usually outperforming women on this task (see for example, [21, 23]).

## Conclusions

Notwithstanding these limitations, to our knowledge, this study is the first investigation of the functioning of topographic memory in OC users. We observed different performances between OC users and non-users, which deserve attention and may help explain the topographic learning mechanisms and sex differences detected in previous studies. Furthermore, the possibility that OC may improve topographic learning skills may lead to applications in women with topographic disorders or visuo-spatial difficulties. Further data on the correlation between topographic skills and hormonal fluctuations are needed to test this interesting hypothesis.

## Abbreviations

CBT: Corsi Block-Tapping Test
DTD: Developmental Topographical Disorientation
FSCS: Familiarity and Spatial Cognitive Style Scale
FSH: Follicle-stimulating Hormone
NCW: Naturally Cycling Women
OC: Oral Contraceptives
WalCT: Walking Corsi Test
